# Substance use amongst individuals with internet gaming disorder and gaming disorder: A scoping review

**DOI:** 10.1556/2006.2025.00170

**Published:** 2026-03-12

**Authors:** Magdalena Liberacka-Dwojak, Christophe Tra, Monika Wiłkość-Dębczyńska, Joëlle Rosselet Amoussou, Marianthi Lousiana Deligianni, Daria Kukuła, Mariya Kuzyan, Jakub Piotrowski, Camilla Sculco, Didier Jutras-Aswad, Yasser Khazaal

**Affiliations:** 1 Faculty of Psychology, Kazimierz Wielki University, Bydgoszcz, Poland; 2 Addiction Medicine Service, Lausanne University Hospital and University of Lausanne, Lausanne, Switzerland; 3 Medical Library-Cery, Lausanne University Hospital and University of Lausanne, Site de Cery, 1008, Prilly, Switzerland; 4 Addiction Medicine, Lausanne University Hospital and University of Lausanne, Lausanne, Switzerland; 5 Institute of Public Health, Università della Svizzera Italiana, Lugano, Switzerland; 6 Department of Psychiatry and Addiction, Université de Montréal, Montreal, Canada; 7 Research Centre, Institut universitaire en santé mentale de Montréal, Montréal, Canada

**Keywords:** internet gaming disorder, substance use, behavioral addiction, scoping review

## Abstract

**Background:**

Internet Gaming Disorder (IGD) and Gaming Disorder (GD) have emerged as significant public health concerns, with studies highlighting their association with substance use. Research on IGD/GD faces challenges due to heterogeneous definitions and measurement tools. While the introduction of DSM-5 and ICD-11 criteria for these behaviors improved research consistency, substance use patterns in individuals with IGD/GD, when defined strictly by these criteria, remain unexplored. Importantly, a comprehensive review of substance use patterns among individuals with IGD/GD based on DSM-5 and ICD-11 criteria has never been conducted.

**Objective:**

This scoping review aims to map existing literature on substance use in individuals with IGD/GD, focusing on patterns, underlying mechanisms, and moderating factors influencing this relationship.

**Methods:**

The review adhered to the JBI manual for scoping reviews and PRISMA-ScR standards. A literature search was conducted in August 2025, in seven bibliographic databases, supplemented by citation tracking strategies. Inclusion criteria encompassed empirical studies published post-2013, using scales published after 2013, based on DSM-5/ICD-11 criteria for IGD/GD, and focusing on substance use.

**Results:**

A total of 36 studies out of 5,561 identified, predominantly cross-sectional, were included. Findings indicated a high co-occurrence of IGD/GD and substance use, particularly among adolescents and young adults. Shared risk factors such as impulsivity, sensation-seeking, and maladaptive coping strategies were identified. Alcohol, tobacco, stimulants, and cannabis emerged as the most commonly used substances, with variations across cultural contexts. Limited longitudinal data underscored the need for research on the progression and interaction of IGD/GD and substance use over time.

**Conclusion:**

The findings revealed that individuals with IGD/GD frequently engage in substance use, including alcohol, tobacco, cannabis, and stimulants such as amphetamines. This review highlights critical mechanisms linking IGD/GD and substance use, emphasizing the role of behavioral reinforcement and emotional dysregulation. Future research should focus on longitudinal designs and protective factors to inform tailored prevention and intervention strategies. Systematic screening for substance use is warranted among individuals with IGD/GD.

## Background

In recent years, changes in Internet access, along with the multiplication of devices and platforms, have rapidly increased the role of technology in daily life and work, and have brought to the forefront important questions regarding the possible negative effects of technology on public health. According to the International Telecommunication Union, 63% of the population worldwide had access to the Internet in 2021 (*ITU Facts and Figures 2021 | Tableau Public*, 2021). While the democratization of Internet access has greatly enhanced productivity and connectivity, it also brings challenges: notably the problematic use of video games, a condition associated with negative educational outcomes (Gentile, 2009; Mihara & Higuchi, 2017) and psychiatric comorbidities such as depression, anxiety, social phobia, ADHD and obsessive-compulsive disorder (González-Bueso et al., 2018).

Following the rapid growth of the gaming industry in the early 2000s (Kuss, 2013), disorders related to video gaming were included in both the Diagnostic and Statistical Manual of Mental Disorders DSM-5 (*Diagnostic and Statistical Manual of Mental Disorders*, 2013) and the International Classification of Diseases ICD-11 (World Health Organization, 2022), respectively as a diagnosis that warrant further research (Internet Gaming Disorder - IGD) and as a formal diagnosis (Gaming Disorder - GD). Its prevalence varies considerably from one country to another, in part due to cultural variations (Brand et al., 2025; Kim et al., 2022; Király, Koncz, Griffiths, & Demetrovics, 2023; Kuss, 2013; Stevens, Dorstyn, Delfabbro, & King, 2021; Strizek et al., 2020) and the heterogeneity in research methodologies (Darvesh et al., 2020; Kim et al., 2022; King, Chamberlain, et al., 2020a; Kuss, 2013; Mihara & Higuchi, 2017; Stevens et al., 2021). Concerns have also been raised about the validity of measurement tools, especially regarding very high prevalence measures (King, Chamberlain, et al., 2020a). Prevalence estimates range from as low as 0.2% to as high as 57.5% (Darvesh et al., 2020), with a metanalysis reporting a pooled prevalence of 3.3% (Kim et al., 2022).

The COVID-19 pandemic has exacerbated the video games use (King, Delfabbro, Billieux, & Potenza, 2020; Ko & Yen, 2020; Xu, Park, Kang, Choi, & Koo, 2021), whereas substance use patterns have shown a more complex and heterogeneous evolution. For example, the use of some psychostimulants such as MDMA temporarily declined during lockdowns, with an upward trend observed after restrictions were lifted (Babicki, 2022; European Monitoring Centre for Drugs and Drug Addiction, 2022; Layman et al., 2022; Mallet, Dubertret, & Le Strat, 2021; Roberts et al., 2021; Xu et al., 2021) Overall, both video games and psychoactive substances have been used as coping mechanisms for stress induced by the pandemic and related social restrictions, although the specific trajectories of use varied across substances(King et al., 2020; Ko & Yen, 2020; Mallet et al., 2021; Xu et al., 2021). Additionally, the co-usage of video games and substances could have a significant clinical impact, as it does in gambling disorder. For example, a systematic review by Markouris et al. identified lower alcohol use among individuals with gambling disorder as a positive prognosis factor for favorable outcomes following psychological treatment (Merkouris, Thomas, Browning, & Dowling, 2016).

Nascent literature looking into this dyad has found a possible association between the occurrences of gaming and substance use (AlSayyari & AlBuhairan, 2018; Andre et al., 2021; Coeffec et al., 2015; Di Carlo et al., 2023; Gallimberti et al., 2016; Munoz-Miralles et al., 2016; Padilla-Walker, Nelson, Carroll, & Jensen, 2010; Ream, Elliott, & Dunlap, 2011; Strizek et al., 2020; VAN Rooij et al., 2014; Walther, Morgenstern, & Hanewinkel, 2012; Wicki, Studer, Marmet, Khazaal, & Gmel, 2025; Škařupová, Blinka, & Ťápal, 2018). Strizek et al. (2020) proposed three hypotheses to explain this association: 1) common etiological, psychosocial and biological factors may underlie both behaviors, 2) substances and gaming may be used synergistically, either to enhance one another or to cope with the adverse effects of the other, and 3) gaming may be negatively associated with substance use, such as alcohol, as increased gaming time may reduce social situations where drinking usually occurs. However, for substances typically consumed at home, such as cannabis or tobacco, gaming and use may co-occur or even reinforce each other (Jobin, Légaré, Lehmann, & Monson, 2025). While the literature on the interplay of gaming and substance use is still in its early stages, a deeper understanding of these interactions is essential to addressing gaming disorder as a multifaceted issue.

### Study rationale

One key rationale for this scoping review is the need to address limitations in prior studies, which often included scales predating the publication of DSM-5 or relied on measurements that lack alignment with current diagnostic criteria (Burleigh, Griffiths, Sumich, Stavropoulos, & Kuss, 2019; King, Chamberlain, et al., 2020b). Previous research (King, Chamberlain, et al., 2020b) noted that research remains divided between studies employing older and the newest measurement tools, creating heterogeneous conclusions. This study was undertaken to update the literature and specifically includes only studies that utilized DSM-5 or ICD-11 criteria, and measurement tools developed after the publication year of the DSM-5. By restricting the scope to such studies, this review seeks to enhance the homogeneity of the data. Given the early stage of research and limited data, a scoping review was chosen to map the existing literature and identify knowledge gaps, as heterogeneity in measurement tools complicates systematic reviews and meta-analyses (King, Chamberlain, et al., 2020).

This scoping review primarily aimed to provide a descriptive overview of substance use patterns amongst individuals with IGD/GD. In this review, substances refer to those not scheduled under international drug control conventions (e.g., alcohol, tobacco, caffeine) and those scheduled under these conventions (e.g., cannabis, stimulants, opioids), while recognizing that national jurisdictions may regulate these substances differently. The secondary research questions were: 1) to examine factors underlying the co-occurrence of gaming and substance use and 2) to determine the moderating factors impacting the strength of this association.

## Methodology

The study protocol was registered on Open Science Framework (https://osf.io/kyf6b) on 25/03/2022.

This scoping review was carried out in accordance with the JBI manual for scoping reviews (Peters et al., 2020). Reporting followed the PRISMA Extension for Scoping Reviews (PRISMA-ScR) guidelines (Tricco et al., 2018).

### Eligibility criteria

**Population**: all individuals diagnosed with IGD or GD based on the DSM-5 or ICD-11 criteria, or assessed using scales based on these criteria, published after 2013. There were no restrictions regarding age, sex/gender, ethnicity, or comorbidity.
**Concept**: primary focus on substance use, with no restrictions on substance type. Gambling and other behavioral addictions were excluded.
**Context**: no restrictions on the context in which the studies were conducted.

### Information sources and search strategy

**Fig. 1.**
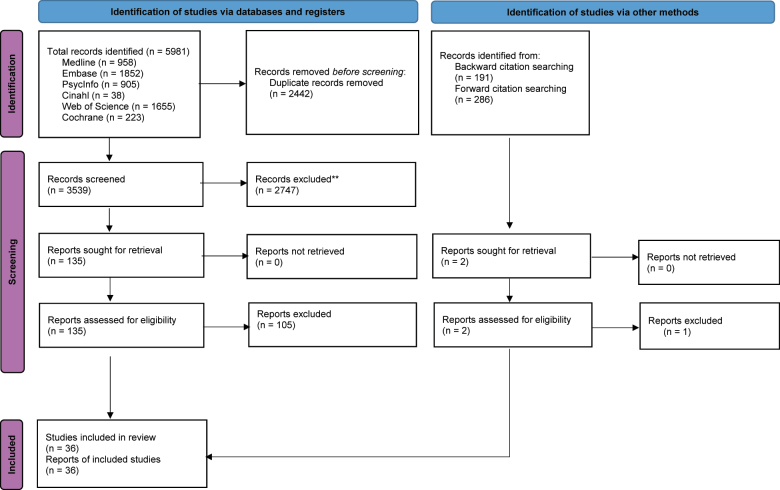
The PRISMA flow diagram for the systematic review detailing the database searches, the number of abstracts screened and the full texts retrieved. *Source:* Page MJ, et al. BMJ 2021; 372:n71. doi: 10.1136/bmj.n71.

A literature search was conducted in August 2025, in collaboration with a medical librarian (JRA), in seven bibliographic databases: Medline ALL Ovid, Embase.com, APA PsycInfo Ovid, CINAHL EBSCO, Web of Science Core Collection, the Cochrane Database of Systematic Reviews Wiley and the Cochrane Central Register of Controlled Trials Wiley. Databases were searched from 2013 onward, without language restrictions. The search equation explicitly included multiple substances of interest, such as alcohol, tobacco, cannabis, and stimulants (e.g., amphetamines), to ensure a comprehensive coverage of the literature. The final search strategies were peer-reviewed by a second information specialist and are documented in the publication's appendix with the search syntax, keywords and index terms used. Additional records were identified through backward and forward citation tracking of included studies, using Citationchaser, ResearchRabbit and the Web of Science Core Collection (Fig. 1).

### Selection of sources of evidence

Studies meeting all the following criteria were included:

- fit into the PCC framework (Population, Concept, and Context),
- published after 2013 to provide a clear definition of IGD/GD for participants selection based on DSM-5/ICD-11 criteria,
- include both qualitative and quantitative studies,
- provide a clear definition of IGD/GD for participants selection based on DSM-5/ICD-11 criteria.

Studies were excluded if they were not based on empirical data (e.g. case reports, protocols, theses, reviews, metanalysis), used scales based on alternative diagnostic criteria (e.g. gambling disorder) adapted to video gaming or published before 2013 or measured substance use by compounding it into a broader context of “problematic behaviors” or “at-risk behaviors”.

### Study selection process

Deduplication of references exported from the databases was performed in Endnote 20 (Clarivate Analytics, USA) (JRA). The selection process was conducted in two stages using Rayyan, a software designed for article selection in systematic reviews: (1) review of titles and abstracts for preliminary inclusion, (2) full-text review using the inclusion and exclusion criteria. Six independent reviewers (CT, CS, MLD, MK, DK, JP) conducted the review at each stage, resolving discrepancies through discussion. Each record was independently screened by two reviewers. Discrepancies were resolved through discussion, with unresolved cases adjudicated by a senior reviewer (YK). The extracted data included the following variables: title, authors, year of publication, country, aims, population/sample size, recruitment procedure, type of study, data collection method, IGD or GD identification method, substances studied, substance use measurement method, follow-up duration, main results, and key findings. The results were synthesized into a narrative review to provide an overview of substance use patterns among individuals with IGD/GD, the mechanisms underlying the association, and the moderating factors influencing the strength of this association. The literature was mapped in terms of types of studies and substances investigated in order to identify knowledge gaps and provide guidance for future research. In addition, the methodological quality of all included studies was assessed using the appropriate JBI Critical Appraisal Checklists (Barker et al., 2023; Munn et al., 2023). Each study was independently evaluated by two reviewers, with discrepancies resolved through discussion and, if necessary, adjudicated by a senior reviewer (YK). This ensured consistency and transparency in the quality appraisal process.

## Results

The systematic search identified 35 studies (see Appendix 1) published between 2013 and 2023, primarily conducted in North America, Europe, and Asia, reflecting cultural differences. Thirty-two studies (90.3%) employed a cross-sectional design. Only three studies (9.7%) used longitudinal designs, providing insights into temporal relationships between IGD and substance use. Sample sizes ranged from 64 participants in clinical settings to 3,939 in large-scale surveys. Most studies targeted adolescents and young adults, ranging from 13 to 30 years. The majority of participants were males, accounting for approximately 70–85% of the samples, reflecting the possible higher prevalence of gaming disorders among males. The appraisal revealed that the majority of cross-sectional studies exhibited moderate-to-high methodological quality. Most used validated self-report instruments, ensuring internal consistency and construct validity, although reliance on self-assessment measures may have introduced response bias, particularly regarding sensitive behaviors such as substance use or gaming. Inclusion criteria and sample characteristics were generally well described, but confounding control was usually limited to demographic variables (e.g., age, sex, education). In contrast, the cohort studies showed somewhat higher methodological rigor, particularly through the use of prospective designs and standardized diagnostic tools. However, they also faced challenges such as attrition bias and limited follow-up duration. Only a minority of studies in both groups applied multivariate adjustments that incorporated psychosocial or contextual factors (see Tables 1 and 2).

**Table 1.** Quality of reporting of the included cross-sectional studies according to JBI critical appraisal tool

	Were the criteria for inclusion in the sample clearly defined?	Were the study subjects and the setting described in detail?	Was the exposure measured in a valid and reliable way?	Were objective, standard criteria used for the measurement of the condition?	Were confounding factors identified?	Were strategies to deal with confounding factors stated?	Were the outcomes measured in a valid and reliable way?	Was an appropriate statistical analysis used?	Overall appraisal
Aonso-Diego et al. (2024)	YES	YES	YES	YES	YES	NO	YES	YES	Include
Ayala-Rojas et al. (2024)	YES	YES	YES	YES	YES	YES	YES	YES	Include
Brandao et al. (2022)	YES	YES	YES	YES	YES	YES	YES	YES	Include
Burkauskas et al. (2022)	YES	YES	YES	YES	YES	YES	YES	YES	Include
Burleigh, Griffiths, Sumich, Wang, Stavropoulos, et al. (2022)	YES	YES	YES	YES	YES	NO	YES	YES	Include
Burleigh, Griffiths, Sumich, Wang, and Kuss (2022)	YES	YES	YES	YES	YES	NO	YES	YES	Include
Byeon et al. (2022)	YES	YES	YES	YES	YES	YES	YES	YES	Include
Di Carlo et al. (2023)	YES	YES	YES	YES	YES	YES	YES	YES	Include
Dowd et al. (2022)	YES	YES	YES	YES	YES	NO	YES	YES	Include
El Fiky et al. (2022)	YES	YES	YES	YES	YES	NO	YES	YES	Include
Gerdner and Hakansson (2022)	YES	YES	YES	YES	YES	NO	YES	YES	Include
Gomez et al. (2022)	YES	YES	YES	YES	YES	NO	YES	YES	Include
Gomez et al. (2023)	YES	YES	YES	YES	YES	NO	YES	YES	Include
Granero et al. (2021)	YES	YES	YES	YES	YES	YES	YES	YES	Include
Turhan Gurbuz et al. (2021)	YES	YES	YES	YES	YES	NO	YES	YES	Include
Horvath et al. (2022)	YES	YES	YES	YES	YES	YES	YES	YES	Include
Ip et al. (2022)	YES	YES	YES	YES	YES	NO	YES	YES	Include
Isralowitz et al. (2022)	YES	YES	YES	YES	YES	YES	YES	YES	Include
Liese et al. (2020)	YES	YES	YES	YES	YES	NO	YES	YES	Include
Liu et al. (2021)	YES	YES	YES	YES	YES	YES	YES	YES	Include
Mallorqui-Bague et al. (2017)	YES	YES	YES	YES	YES	YES	YES	YES	Include
Mannikko et al. (2020)	YES	YES	YES	YES	YES	YES	YES	YES	Include
Mbarki et al. (2023)	YES	YES	YES	YES	YES	YES	YES	YES	Include
Mills et al. (2020)	YES	YES	YES	YES	YES	YES	YES	YES	Include
Mitchell et al. (2021).	YES	YES	YES	YES	YES	YES	YES	YES	Include
Moreno et al. (2022)	YES	YES	YES	YES	YES	YES	YES	YES	Include
Paik et al. (2017)	YES	YES	YES	YES	YES	YES	YES	YES	Include
Sanchez-Llorens et al. (2021)	YES	YES	YES	YES	YES	YES	YES	YES	Include
Seproo et al. (2022)	YES	YES	YES	YES	YES	YES	YES	YES	Include
Singh et al. (2021)	YES	YES	YES	YES	YES	NO	YES	YES	Include
Stavropoulos and Zarate (2022)	YES	YES	YES	YES	YES	NO	YES	YES	Include
Sucha et al. (2024)	YES	YES	YES	YES	YES	YES	YES	YES	Include
Wang (2022)	YES	YES	YES	YES	YES	YES	YES	YES	Include

**Table 2.** Quality of reporting of the included cohort studies according to JBI critical appraisal tool

	Were the two groups similar and recruited from the same population?	Were the exposures measured similarly to assign people to both exposed and unexposed groups?	Was the exposure measured in a valid and reliable way?	Were confounding factors identified?	Were strategies to deal with confounding factors stated?	Were the groups/participants free of the outcome at the start of the study?	Were the outcomes measured in a valid and reliable way?	Was the follow up time reported and sufficient to be long enough for outcomes to occur?	Was follow up complete?	Were strategies to address incomplete follow up utilized?	Was appropriate statistical analysis used?	Overall appraisal
Borges et al. (2023)	YES	YES	YES	YES	YES	NO	YES	NO	NO	YES	YES	Include
Borges et al. (2024)	YES	YES	YES	YES	YES	YES	YES	YES	NO	YES	YES	Include
Wartberg et al. (2025)	YES	YES	YES	YES	YES	NO	YES	YES	YES	YES	YES	Include

Various diagnostic tools were employed to assess IGD, with the Internet Gaming Disorder Scale (IGDS) (Pontes & Griffiths, 2017) being the most commonly used, which was used in 28 studies (80.0%). Some studies employed diagnostic criteria from the DSM-5 and ICD-11 (used in 8 studies; 23.0%). For substance use, a variety of tools were employed, with: The Alcohol Use Disorder Identification Test (AUDIT; Saunders, Aasland, Babor, De La Fuente, & Grant, 1993) being most frequently used for alcohol consumption (used in 15 studies; 42.9%), The Cigarette Dependency Scale (CDS-5; (Etter, Le Houezec, & Perneger, 2003) for nicotine (used in 6 studies; 17.1%), the Drug Abuse Screen Test-10 (DAST; Skinner, 1982) for drugs (used in 6 studies, 17.1%), and self-reported frequency and intensity of use for cannabis and other stimulants which was used in 12 studies (34.2%) (see Table 3). Data collection methods predominantly involved self-reported surveys and standardized questionnaires, often administered online. Alcohol use was the most frequently examined behavior. 15 studies (42.9%) employed the Alcohol Use Disorders Identification Test (AUDIT), which primarily identifies risky drinking patterns. Other studies relied on single-item measures capturing frequency or quantity of alcohol consumption. A smaller subset of studies (*n* = 3; 13.6%) specifically examined the co-occurrence of alcohol use during gaming sessions, indicating simultaneous engagement in both behaviors. Thus, the operationalization of alcohol use measurement varied across studies, ranging from general consumption to risky patterns and simultaneous gaming-related drinking.

**Table 3.** Included articles

Study and origin	Aims	Sample	Type of study	IGD identification method	Substance use identification method	Main results
Aonso-Diego, Gonzalez-Roz, Weidberg, and Secades-Villa (2024); Spain DOI: 10.1016/j.jad.2024.08.203	Identify profiles of young adult gamers based on anxiety, depression, and stress, and examined their differences in gaming severity, gambling, and substance use	*N* = 1,186; young adult gamers	Cross-sectional	IGDS9-SF^1^	HSI^2^ CUDIT-R^3^ BYAACQ^4^	Latent profile analysis identified three gamer profiles: low (*n* = 660), moderate (*n* = 377), and high emotional distress (*n* = 172). Higher emotional distress was associated with greater IGD severity (*F*(2) = 14.671, *p* < .001, *d* = 0.314), increased tobacco use (*χ*^2^(2) = 20.432, *p* < .001, *Φ* = 0.131), illegal substance use (*χ*^2^(2) = 6.782, *p* = .034, *Φ* = 0.076), and greater negative alcohol-related consequences (*F*(2) = 15.248, *p* < .001, *d* = 0.464)
Ayala-Rojas et al. (2024); Spain DOI: 10.1007/s11469-024-01386-x	Estimate the prevalence of online gaming disorder (OGD) and sports betting addiction among patients in behavioral addictions department in hospitals	*N* = 108 with online gaming disorder	Cross-sectional	Semi-structured interview based on DSM-5 criteria	Self-reported substance use	Among individuals with IGD, tobacco use was reported by 22.2%, alcohol use by 1.9%, and other illicit drug use by 3.7%
Borges et al. (2023); Mexico DOI: 10.3390/ijerph20032063	Evaluate if IGD* in the first year of university predicts mental disorders a year later, controlling for baseline mental health disorders (with alcohol and drug use disorders) and demographics	*N* = 1,731; first-year university students	Prospective cohort study with follow-up period of one year	DSM-5 IGD scale^5^	CIDI-SC^6^ for drugs AUDIT^7^	No significant longitudinal impact of IGD on psychiatric disorders and substance use disorder , with risk ratios (RR) close to 1 across outcomes, such as substance use disorders (RR = 1.01, 95% CI [0.95, 1.08])
Borges et al. (2024); Mexico DOI: 10.47626/1516-4446-2024-3816	Evaluate if a wide range of baseline mental disorders predict IGD one to three years later, among university students	*N* = 2,144; first-year university student	Prospective cohort study with follow-up periods ranging from 1 to 3 years	DSM-5 IGD scale^5^	CIDI-SC^6^ for drugs AUDIT^7^	Significant associations of baseline major depressive disorder (HR = 1.98, 95% CI [1.11, 3.54]) and bipolar disorder (HR = 2.60, 95% CI [1.07, 6.32]) with the incidence of IGD. Drug abuse/dependence was also associated (HR = 2.60, 95% CI [1.26, 5.38]) in bivariate analysis but lost significance in multivariate models
Brandao et al. (2022); Brasil DOI: 10.1080/10550887.2021.1971941	Describe the prevalence of the non-problematic vs problematic use of video games among adolescents and assess factors associated with both types of video games	*N* = 3,939; eight-grade students	Cross-sectional	Nine-item dichotomous DSM-5 scale	Self-reported last-year use of alcohol and tobacco	IGD was more frequent in males (95.54%, aOR = 3.43) and the upper class (44.42%), with higher bullying rates (perpetration: 42.63%, victimization: 36.23%) and substance use (alcohol cOR = 1.64, tobacco cOR = 20.09). Predictors included male sex (aOR = 7.08), younger age (aOR = 0.79), and bullying perpetration (aOR = 1.48)
Burkauskas et al. (2022); Multi-country among Europe, Asia and South America DOI: 10.1186/s43045-022-00180-6	Explore the association between IGD, anxiety depressive symptoms, and substance use within young Internet users	*N* = 3,529; college students	Cross-sectional	ICMH-IGD^8^	Self-reported past-month use of alcohol, depressant drugs, marijuana, stimulant drugs, and hallucinogens	In males, Internet browsing (*β* = 0.14), anxiety (*β* = 0.09), and depression (*β* = 0.17) explained 16.1% of IGD variance; in females, Internet browsing (*β* = 0.13), marijuana use (*β* = 0.10), and depression (*β* = 0.21) explained 17.6%. Depressants and hallucinogens were linked to IGD severity in males and stimulants and hallucinogens in females
Burleigh, Griffiths, Sumich, Wang, Stavropoulos, et al. (2022); New Zealand DOI: 10.3390/ijerph192316078	Identify profiles of disordered behaviors, personality traits, co-occurrence, and coping strategies across countries	*N* = 1,916; students	Cross-sectional	IGDS9-SF^1^	CDS^9^ AUDIT^7^ DAST^10^	High-risk profiles exhibited elevated problematic substance use and behavioral addiction scores (*F*[36, 1,098] = 3.93, *p* < 0.001, *η*^2^ = 0.114). Low emotional stability (*z* = −0.39 to −0.81) and dysfunctional coping (*z* = 0.61–1.00) were stable across risk profiles. Extraversion was higher in at-risk profiles (*z* = 0.01 to −0.30), and conscientiousness was lowest in high-risk groups (*z* = −0.53 to −1.30)
Burleigh, Griffiths, Sumich, Wang, and Kuss (2022); Australia, New Zealand, UK DOI: 10.3390/jcm11247370	Compare problematic behaviors and problematic substance use in clinical and non-sub-clinical gamers vs. nongamers, and examine how coping strategies influence gaming and co-occurring issues	*N* = 64 in clinical group and *N* = 138 in non-clinical group	Cross-sectional	IGDS9-SF^1^	CDS^9^ AUDIT^7^ DAST^10^	Gamers were more likely to have problematic substance use-PSU- (*χ*^2^(1, *n* = 64) = 1.01, *p* = 0.04), with people with PSU scoring higher on IGD than people with single substance use (*M* = 24.09 vs. M = 15.6, *t*(28.25) = 3.42, *p* < 0.01). IGD scores were linked to coping styles in non-clinical gamers (*F*(3,83) = 3.61, *p* = 0.01, *R*^2^ = 0.080. Addiction scores were found between non-clinical and poly-substance gamers (*F*(8,134) = 4.23, *p* < 0.001)
Byeon et al. (2022); Korea DOI: 10.1016/j.jad.2021.10.064	Reveal if risky (game addiction) and usual game use (non-risky) are associated with mental health in early adulthood	*N* = 415; young adults	Cross-sectional	GSO-Q^11^	CIDI^2^ for alcohol and nicotine	Individuals with mental disorders, including major depressive disorder, bipolar disorder, drug abuse/dependence, binging/purging, intermittent explosive disorder, and psychotic experiences, had higher IGD rates (22.09 vs. 9.26 per 1,000 person-years). Multivariate analysis showed mental disorders increased IGD risk (HR = 2.33), with differences emerging after the first year. No significant link was found between IGD and substance use disorders
Di Carlo et al. (2023); Italy DOI: 10.1159/000529544	Investigate the association between high-risk gaming and substance use among young adults and explore the psychopathological correlates of high-risk gaming	*N* = 913; young adults	Cross-sectional	IGDT-10^12^	Self-reported lifetime use of different drugs	High-risk gamers had higher rates of lifetime substance use, frequently used cannabinoids, LSD/mushrooms, and poppers, and 32.5% reported frequent or daily psychoactive substance use. They used substances more often, except nicotine and alcohol
Dowd et al. (2022); Australia DOI: 10.1007/s11469-022-00862-6	Examine cross-addiction risk profiles and identify profiles, describe their characteristics and proportions, analyze links to COVID-19-related anxiety, and compare profiles with diagnosable behaviors	*N* = 968; general population	Cross-sectional	IGDS9-SF^1^	AUDIT^7^ DAST^10^ CDS^9^	Two cross-addiction profiles were found: high-risk (42.6%) and low-risk (57.4%). High-risk individuals scored higher on substance abuse and behavioral addictions, with 20.2% exceeding diagnostic cut-offs (vs. 3.8% in low-risk) and reported higher COVID-19 anxiety. Behavioral addictions showed greater convergence than substance abuse across groups
El Fiky et al. (2022); Egypt DOI: 10.1186/s43045-022-00219-8	Determine the rate of IGD among adolescent students and, relations of problematic Internet use with risk factors	*N* = 248; adolescents students	Cross-sectional	IGD^13^	Mini KID^14^ for alcohol and other substances	Mild and moderate Internet addiction affected 42.3 and 35.1% of students, respectively, with 31.5% identified as risky gamers. Psychiatric disorders (major depression and generalized anxiety) were highly prevalent among disordered gamers (92.3%). 4.4% students suffered from alcohol dependence
Gerdner and Hakansson (2022); Sweden DOI: 10.1186/s12888-022-04218-1	Estimate the prevalence and comorbidity of SUDs**, gambling, and gaming disorders in late adolescents using ICD/DSM criteria	*N* = 949; students	Cross-sectional	IGDS^15^	AUDIT^7^ DUDIT^16^ SURNC^17^ ADDIS^18^ SUDDS^19^	SUD was found in 14.6%, more common in girls, primarily involving alcohol, with 26.7% having another psychiatric disorder. Among girls, 40% with SUD had a psychiatric disorder, while 28% with a psychiatric disorder also had SUD. In boys, 22% with SUD had a psychiatric disorder, and 15% with a psychiatric disorder also had SUD
Gomez, Stavropoulos, Brown, and Griffiths (2022); Australia DOI: 10.1016/j.psychres.2022.114605	Examine the factor structure of substance addictions (alcohol, cigarettes, substance use) and behavioral addictions (sex, social media, shopping, exercise, gambling, gaming, internet use)	*N* = 839; adults	Cross-sectional study	IGDS9-SF^1^	AUDIT^7^ DAST^10^ CDS^9^	Behavioral and psychoactive substance addictions were linked to low conscientiousness, agreeableness, and emotional stability (*β* = −0.204 to −0.178). Psychoactive substance addictions were associated with extraversion, agreeableness, conscientiousness, and emotional stability (*β* = −0.294 to 0.179). Both addictions are associated with depression, anxiety, and avoidant coping (*β* = 0.108–0.623)
Gomez, Brown, Tullett-Prado, and Stavropoulos (2023); Australia DOI: 10.1007/s11469-022-00995-8	Examine the network properties of a model involving addictions: alcohol, smoking, drugs, sex, social media, shopping, exercise, gambling, gaming, and internet use	*N* = 968; general population	Cross-sectional	IGDS9-SF^5^	AUDIT^7^ DAST^10^ CDS^9^	Internet use had the highest centrality, followed by gaming (Strength = 1.12) and gambling. Cigarette smoking (Strength = −1.10) and drugs use (Strength = −0.27) ranked among the lowest. Gaming was strongly linked to internet use and moderately to alcohol and drugs
Granero et al. (2021); Spain DOI: 10.1007/s10899-021-10079-2	Use network analysis to explore correlations between sociodemographic and clinical factors in patients with gaming disorder	*N* = 117; patients at the hospital	Cross-sectional	DSM-5 IGD scale^5^	AUDIT^7^ DUDIT^16^	Substance use, with alcohol being the most common, is linked to IGD. Key factors included self-transcendence, cooperativeness, emotional distress, and age of onset. Cluster is linked to impulsivity, novelty seeking, and tobacco use, while another is tied to emotional distress, alcohol use, and harm avoidance. Comorbidities included behavioral addictions (37.6%), alcohol use (23.1%), and tobacco use (25.6%)
Turhan Gurbuz et al. (2021); Turkey DOI: 10.1080/10826084.2021.1958856	Investigate IGD, social media addiction, and loneliness levels in adolescents and youths with substance use issues	*N* = 184; adolescents and young adults	Cross-sectional study	IGDS9-SF^12^	DUDIT^16^	Adolescents with problematic gaming had higher loneliness (*M* = 44.08 vs. 36.06, *p* < 0.001) than controls. Adolescents with SUD had lower IGD scores than the ones without SUD (*M* = 13.40 vs. 15.60, *p* = 0.032)
Horvath et al. (2022); Hungary DOI: 10.1159/000517042	Investigate the association of alcohol and illicit drug use with gaming disorder severity	*N* = 2,768; 9th and 11th grade adolescents	Cross-sectional	IGDT-10^12^	Self-reported lifetime and past-month use of alcohol, cannabis, amphetamines, and medications for non-medical purposes	Substance users showed higher GD severity and the highest rates of alcohol and illicit drug use. They had greater odds of giving up activities and facing negative consequences (OR = 20.48, *p* < 0.001). Gaming frequency among this group may reflect substance use as a coping mechanism, influenced by gender and school grade
Ip et al. (2022); USA DOI: 10.1016/j.chb.2021.106890	Determine the prevalence of performance-enhancing drug use and IGD among adult gamers and identify IGD risk factors	*N* = 526; adults	Cross-sectional	IGDS9-SF^1^	Self-reported past month's use of performance-enhancing drugs including caffeine drinks, energy drinks, prescription stimulants, modafinil	Approximately 42.6% of gamers used PEDs, with energy drinks (33.8%) and caffeine drinks (19.2%) being the most common. Prescription drugs were used by 5.4% of gamers to enhance performance; 4.9% used a prescription stimulant. IGD criteria were met by 32.3% of gamers, with associated factors including ADHD diagnosis (OR 3.5) and prescription PED use (OR 5.5)
Isralowitz et al. (2022); Switzerland DOI: 10.1007/978-3-319-41556-7_4	Examine the impact of gaming and gaming disorder on the wellbeing of male university students and other adults	*N* = 526; adults	Cross-sectional	IGDS9-SF^1^	SUSI^20^ for tobacco, alcohol, cannabis, Ritalin, pain relievers, sedatives	Last-month substance use, particularly alcohol, was high (75.3%). Individuals with severe gaming disorder exhibited significant substance abuse, including alcohol, tobacco, cannabis, and sedatives, potentially linked to mental health deterioration, burnout, and economic factors
Liese, Kim, and Hodgins (2020); USA DOI: 10.1016/j.addbeh.2020.106432	Test if emotion dysregulation mediates the link between insecure attachment and substance use disorder/behavioral dependencies	*N* = 689; students	Cross-sectional	Self-reported DSM-5 symptoms	Self-reported lifetime use of alcohol and marijuana-based on DSM-5 criteria	Significant differences were found based on age, *F*(3, 688) = 3.35, *p* < 0.05, with younger participants being more addicted to the Internet (*M* = 19.45, SD = 2.202). More males chose marijuana (53.1%). There were no differences between anxious attachment and IGD, *F*(1, 336) = 2.44, *p* = .231; avoidant attachment, *F*(1, 336) = 3.66, *p* = .059; and IGD
Liu et al. (2021); China DOI: 10.1016/j.adolescence.2021.06.008	Determine the prevalence rate and associated factors of IGD among adolescents and examine the predictors for IGD incidence over one year	*N* = 1,121; adolescents	Longitudinal study with a follow-up period of one year	IGDS^15^	Self-reported lifetime use of tobacco, alcohol, heroin, MDMA, marijuana, crystal meth, morphine, and other drugs	IGD was significantly associated with substance use, including tobacco, binge drinking, and illicit drugs, in cross-sectional analysis, but substance use did not predict IGD incidence longitudinally
Mallorqui-Bague et al. (2017); Spain DOI: 10.1556/2006.6.2017.078	Study the co-occurrence of IGD and online gambling disorder	*N* = 228; adults	Cross-sectional study	Semi-structured face-to-face clinical interview based on DSM-5 criteria	YFAS-S^21^ for tobacco, alcohol, and drugs	IGD patients have lower tobacco consumption (|*d*| = 0.63, *p* = 0.004) as well as higher Body Mass Index (|*d*| = 0.55, *p* = 0.044) and YFFAS-S (|*d*| = 0.51, *p* = 0.038) scores compared with the online GD group. IGD patients are younger, more likely to be single and unemployed, and have higher emotional distress, harm avoidance, and reward dependence traits
Mannikko, Ruotsalainen, Tolvanen, and Kaariainen (2020); Finland DOI: 10.1007/s11469-019-00100-6	Examine the connections between problematic online gaming and various health-related behavior characteristics	*N* = 773; students	Cross-sectional study	IGDT-10^16^	Self-reported lifetime use of tobacco, snuff, alcohol, and drugs	Problematic gaming was reported by 35.3% of students, with higher prevalence in boys (54.4% vs. 22.8%, *p* < 0.001). Substance use was noted in 10.1% for alcohol and 7.0% for drugs, with cannabis being the most common (4.4%). Boys were more likely to use substances than girls (*p* < 0.001). The co-occurrence of IGD and substance use was linked to male sex (aOR = 3.37, *p* < 0.001), younger age (aOR = 1.9, *p* = 0.007), depression, anxiety, and alexithymia
Mbarki et al. (2023); Tunisia DOI: 10.26719/emhj.23.121	Assess the prevalence of addiction problems and co-occurrences of addictive behaviors, and disorders among high school students	*N* = 1,399; high school students	Cross-sectional	Questionnaire based on DSM-5 criteria for IGD	Self-reported past month's use of cigarette, e-cigarette, tobacco, alcohol, drugs	Problematic gaming was reported by 35.3% of students, while 7.0% used drugs and 10.1% consumed alcohol. Boys had higher rates of both gaming addiction and substance use (*p* < 0.001). The co-occurrence of IGD and substance use was linked to male sex (aOR = 217.004, *p* < 0.001), grade repetition (aOR = 0.232, *p* < 0.001), depression (aOR = 0.232, *p* < 0.001), anxiety (aOR = 0.335, *p* = 0.003), and alexithymia (aOR = 0.361, *p* = 0.005)
Mills, Marchica, Keough, and Derevensky (2020); USA, Canada DOI: 10.1080/14459795.2020.1752768	Compare substance use among emerging adults at-risk for problem gambling and/or video gaming	*N* = 1,621; young adults	Cross-sectional study	IGDS9-SF^1^	ASSIT^22^	Video gaming classifications were linked to substance use, with At-Risk gamers reporting more frequent drug use (*χ*^2^(4) = 33.05, *p* < .001) and less alcohol use. Frequent gaming slightly reduced cigarette, alcohol, and drug use (ORs 0.98–0.99). At-Risk PVG was associated with higher occasional (OR = 2.32) and frequent drug use (OR = 3.63), while At-Risk DC showed stronger links to frequent cigarette (OR = 3.78) and drug use (OR = 3.98–18.87)
Mitchell et al. (2021); USA DOI: 10.1007/s11469-021-00643-7	Explore group differences in electronic nicotine delivery systems and GD/IGD dependency and the impact of ADHD symptoms on their association	*N* = 1,054; students	Cross-sectional study	CSAS^23^	EDS^24^	GD/IGD symptoms were linked to greater EDS dependency scores (*r* = 0.174, *p* < 0.001), with men more likely than women to show concurrent GD/IGD and e-cigarette dependency (*χ*^2^(1, *N* = 1,054) = 17.69, *p* < 0.001). ADHD symptoms significantly moderated this relation (Δ*R*^2^ = 0.067, *F*(1, 1036) = 24.75, *p* < 0.001), strengthening the connection between GD/IGD and e-cigarette dependency
Moreno et al. (2022); USA DOI: 10.2196/27719	Examine problematic Internet use, IGD, and social media addiction together	*N* = 6,000; young adults	Cross-sectional study	IGD^13^	AUDIT^7^	Problematic alcohol use was positively associated with IGD (OR = 1.26, 95% CI [1.09, 1.44], *p* < 0.05). IGD was linked to sleep issues (OR = 4.6, 95% CI [2.9, 7.2]) and severe anxiety (OR = 5.2, 95% CI [2.5, 10.9]). Anxiety had a weaker relation to IGD than depression
Paik et al. (2017); South Korea DOI: 10.3390/ijerph14121512	Explore gaming behaviors and clinical characteristics across different gaming device usage patterns and their role in IGD	*N* = 3,058; adults	Cross-sectional study	Self-reported DSM-5 symptoms for IGD	AUDIT^7^ FTND^25^	Alcohol Use Disorder (AUD) and nicotine dependence were more prevalent in people with IGD (AUD: *χ*^2^ = 75.003, *p* < 0.001; nicotine: *χ*^2^ = 25.675, *p* < 0.001). Nicotine dependence was higher in IGD males, while AUD was more common in IGD females. PC gaming was associated with higher symptoms of IGD than smartphone gaming
Sanchez-Llorens et al. (2021); Spain DOI: 10.20882/adicciones.1629	Identify personality traits associated with GD in adolescents, explore links between GD, SUD, psychopathology, and academics, and inform prevention programs for at-risk personality types	*N* = 119; students	Cross-sectional study	GASA^26^ CERV^27^	AUDIT^7^ CRAFFT^28^ POSIT^29^	GD was significantly associated with school maladjustment (OR = 1.08, 95% CI [1.00–1.17], *p* = .047) and male gender (OR = 4.82, 95% CI [1.17–19.81], *p* = .029). SUD in individuals with GD was linked to school maladjustment (OR [95% CI] = 1.06 [1–1.13]; *p* = . 048), neuroticism (OR = 1.07, 95% CI [1.00–1.14], *p* = .040), and clinical maladjustment (OR = 1.10, 95% CI [1.01–1.20], *p* = .020)
Seproo et al. (2022); Iran DOI: 10.34172/jrhs.2022.91	Subgroup students based on risk-taking behavior and investigate the independent role of IGD in the membership of participants in each latent class	*N* = 1,294; university students	Cross-sectional	IGDS9-SF^1^	A WHO-based questionnaire (Amin-Esmaeili et al., 2016) assessed substance use frequency (yes/no) over the last month, year, and lifetime	Three risk classes were identified: low-risk (fights: 13%), tobacco smokers (smoking: 78%, hookah: 88%), and high-risk (smoking: 80%, hookah: 62%, alcohol: 81%, drugs: 55%). Being single (OR = 2.28, CI: 1.19–4.37) and unemployment increased the odds for tobacco smokers (OR = 1.56, CI: 1.04–2.33) and high-risk (OR = 1.43, CI: 1.11–1.84) groups. Alcohol use was 58% higher in the high-risk group. IGD scores did not predict class membership
Singh, Mani Pandey, Datta, and Batra (2021); India DOI: 10.1016/j.cegh.2021.100885	Compare stress, internet use, substance use, and coping among adolescents, young adults, and middle-aged adults	*N* = 10,27l young adults and middle-aged adults	Cross-sectional study	IGDS9-SF^1^	Self-reported lifetime use of alcohol, tobacco, and self-medication use based on DSM-5 criteria	Adolescent females reported the highest stress (*M* = 20.91) and IGD scores (*M* = 52.59). Young adult and middle-aged males showed higher substance use (*M* = 1.50; *M* = 1.17) and maladaptive coping (*M* = 10.83; *M* = 10.03). Gender and age significantly influenced the relationship between stress, IGD, and substance, with males and younger individuals more affected
Stavropoulos and Zarate (2022); USA, UK, Australia, New Zealand DOI: 10.1016/j.abrep.2022.100406	Conduct a network analysis of symptoms of different forms of addictive behaviors to examine the behavioral commonalities and interrelations	*N* = 839; adults	Cross-sectional study	IGDS9-SF^1^	AUDIT^7^ DAST^10^ CDS^9^	IGD is linked to drug use (*r* = 0.105), driven by shared symptoms and reciprocal cycles. Depression, anxiety, maladaptive coping, and adverse experiences contribute to these patterns
Sucha et al. (2024); Czech Republic DOI: 10.1556/2006.2024.00045	Estimate the association between IGD, substance use, and other risky behaviors in adolescents	*N* = 3,950; adolescents	Cross-sectional	IGDS9-SF^1^	SRBA^30^	The prevalence of IGD was 3.62% (95% CI [3.1, 4.3%]), higher in males (5.89%) than females (1.45%). People with IGD showed increased use of pharmaceuticals, MDMA, and LSD, while non-gamers had higher alcohol, cigarette, sedative, and marijuana use. A U-shaped relationship between gaming and alcohol/cigarette consumption was observed, with non-disordered gamers reporting the lowest substance use rates
Wang (2022); China DOI: 10.1007/s11469-022-00866-2	Explore the link between alcohol consumption and gaming	*N* = 1,118; adults	Cross-sectional study	IGDT-10^12^	AUDIT^7^	Daily gamers scored higher on AUDIT (*M* = 7.89 vs. 7.13, *p* = 0.023) and IGDT-10 (*M* = 7.51 vs. 5.85, *p* < 0.001) than weekly gamers, with a greater likelihood of meeting criteria for internet gaming disorder (5.9% vs. 1.3%, *p* < 0.001) and alcohol use disorder (43.9% vs. 36.6%, *p* = 0.015). IGDT-10 severity scores were linked to harmful alcohol consequences, with a stronger association in females
Wartberg et al. (2025) DOI: 10.3390/ejihpe15050077	To examine reciprocal longitudinal relations between the Big Five personality traits and problematic use of video games, social media, and alcohol, and to test cross-behavioral influences	*N* = 443	Longitudinal study with a follow-up period of three years	IGDS^15^	AUDIT^7^	IGD and problematic alcohol use were positively correlated (*r* = .13–.21, *p* < .01). Both were linked to low conscientiousness (IGD *r* = −.31; AUDIT *r* = −.15 to −.19, *p* < .01) and low agreeableness (IGD *r* = −.24 to −.31; AUDIT *r* = −.14 to −.25, *p* < .01), while IGD also related to low Extraversion and AUDIT to higher negative emotionality

*IGD: Internet gaming disorder.

**SUD: Substance use disorder.

^1^IGDS9-SF: Nine-Item Internet Gaming Disorder Scale-Short Form (Pontes & Griffiths, 2017).

^2^HSI: Heaviness of Smoking Index (Borland, Yong, O’Connor, Hyland, & Thompson, 2010).

^3^CUDIT-R: Cannabis Use Disorder Identification Test (Adamson et al., 2010).

^4^BYAACQ: Brief Young Adult Alcohol (Kahler, Hustad, Barnett, Strong, & Borsari, 2008).

^5^DSM-5 IGD scale: 23-item instrument developed for the World Health Organization's World Mental Health Surveys (Borges et al., 2019).

^6^CIDI-SC: The Composite International Diagnostic Interview Screening Scales (Kessler et al., 2013).

^7^AUDIT: The Alcohol Use Disorders Identification Test (Saunders et al., 1993).

^8^ICMH-IGD: The International Child Mental Health Study Group (Stevanović et al., 2020).

^9^CDS: Cigarette Dependency Scale-5 (Etter et al., 2003).

^10^DAST: Drug Abuse Screen-Test (Skinner, 1982).

^11^GSO-Q: Game Overuse Screening Questionnaire (Baek et al., 2020).

^12^IGDT-10: Ten-Item Internet Gaming Disorder Test (Király et al., 2019).

^13^IGD: The Internet Gaming Disorder Scale (Lemmens, Valkenburg, & Gentile, 2015).

^14^MINI KID: The Mini International Neuropsychiatric Interview for children and adolescents (Sheehan et al., 2010).

^15^IGDS: Internet Gaming Disorder Scale (Pontes & Griffiths, 2017).

^16^DUDIT: Drug Use Disorders Identification Test (Berman, Wennberg, & Källmén, 2012).

^17^SURNC: Substance Use Related Negative Consequences (Hagborg, Thorvaldsson, & Fahlke, 2020).

^18^ADDIS: Alkohol Drog Diagnos InStrument (Gerdner & Hakansson, 2022).

^19^SUDDS: Substance use disorder diagnostic schedule(Gerdner & Hakansson, 2022).

^20^SUSI: Center's Substance Use Survey Instrument (Isralowitz, Afifi, & Reznik, 2016).

^21^YFAS-S: Yale Food Addiction Scale (Saffari et al., 2022).

^22^ASSIT: Modified version of Alcohol, Smoking, and Substance Involvement Screening Test (ASSIST) (*The Alcohol, Smoking and Substance Involvement Screening Test (ASSIST)*, n.d.).

^23^CSAS: Video Game Dependency Scale (Rehbein, Kliem, Baier, Mößle, & Petry, 2015).

^24^EDS: E-cigarette Dependence Scale (Morean, Krishnan-Sarin, & S. O’Malley, 2018).

^25^FTND: Fagerstrom Test for Nicotine Dependence (Heatherton, Kozlowski, Frecker, & Fagerström, 1991).

^26^GASA: The Game Addiction Scale for Adolescents (André, Munck, Håkansson, & Claesdotter-Knutsson, 2022).

^27^CERV: The Questionnaire of Experiences Associated with Video games (Chamarro et al., 2014).

^28^CRAFFT: Abuse Screening Test (Rial et al., 2019).

^29^POSIT: Problem Oriented Screening Instrument for Teenagers (*Problem Oriented Screening Instrument for Teenagers | Www.Euda.Europa.Eu*, n.d.).

^30^SRBA: The scale of risk behavior in adolescents (Dolejš, Skopal, & Suchá, 2019).

The main objectives of the included studies aligned with the rationale of this review, focusing primarily on the prevalence of substance use among individuals with IGD/GD, the examination of co-occurrence patterns, and the identification of associated psychosocial and personality factors.

### Substance use patterns

Studies explored the use of various substances, including alcohol, tobacco, cannabis, and stimulants such as amphetamines. Tobacco use was particularly prevalent among adolescents and young adults, often reported as a parallel behavior with gaming ranging from 20.09% to 78–88% among high-risk groups (Brandao, Sanchez, de, & da Silva Melo, 2022; Burleigh, Griffiths, Sumich, Wang, Stavropoulos, et al., 2022; Di Carlo et al., 2023; Seproo et al., 2022). Alcohol use was widespread, and often linked to cultural acceptance and accessibility, with prevalence rates ranging from 43.9 to 75.3% (Borges et al., 2023; Brandao et al., 2022; Isralowitz et al., 2022; Wang, 2022). Stimulant use, such as amphetamines, was less frequently studied but observed in certain contexts, especially among competitive gamers. This group often used stimulants to prolong gaming sessions or enhance performance with prevalence around 4.9% (Horvath et al., 2022; Ip, Urbano, Caballero, Lau, & Barnett, 2022). Polysubstance use, involving combinations of alcohol, tobacco, and cannabis, was particularly reported in clinical samples of individuals with IGD/GD, especially in those with psychiatric comorbidities (Burleigh, Griffiths, Sumich, Wang, Stavropoulos, et al., 2022; Di Carlo et al., 2023). Polysubstance use was assessed in four studies (18.2%). Reported prevalence rates ranged from 12 to 28% of IGD/GD participants, depending on the sample and measurement approach.

### Shared risk factors

Studies indicate that IGD and substance use can be particularly linked in clinical populations, where impulsivity and emotional dysregulation play a significant role (Ayala-Rojas et al., 2024; Sucha, Dolejs, Dostal, Pipova, & Pontes, 2024). Other studies noted significant similarities between IGD and substance use, suggesting impulsivity, sensation-seeking, and coping strategies for stress as risk factors (Burleigh, Griffiths, Sumich, Wang, Stavropoulos, et al., 2022; Gerdner & Hakansson, 2022). These traits were especially significant among adolescents engaging in problematic gaming, who were more likely to use substances such as cannabis and synthetic drugs, highlighting their vulnerability to addictive behaviors (Di Carlo et al., 2023). Moreover, a higher prevalence of maladaptive coping strategies, such as substance use to manage emotional dysregulation, was highly evident in this group (Burleigh, Griffiths, Sumich, Wang, Stavropoulos, et al., 2022; Horvath et al., 2022). Moreover, research suggest that anxiety and depression can increase the co-occurrence of addictive behaviors (Mbarki et al., 2023).

Furthermore, a few moderating factors were highlighted in the studies, with age and gender being the most significant. These differences indicate that males and younger individuals are more likely to show associations between gaming and substance use, a relationship shaped by social interactions and sensation-seeking behaviors (Borges et al., 2023; Granero et al., 2021). In addition, personality traits, such as neuroticism, impulsivity, and sensation-seeking emerged as relevant traits moderating this association (Burleigh, Griffiths, Sumich, Wang, Stavropoulos, et al., 2022; Paik, Cho, Chun, Jeong, & Kim, 2017). Similarly, clinical groups exhibited higher rates of substance use, including ADHD which also involves traits such as impulsivity and sensation-seeking, along with depression, and anxiety, suggesting cross-addiction mechanisms (Borges et al., 2023; Granero et al., 2021). A few studies also indicated that single individuals were at higher risk of engaging in both IGD and substance use, potentially due to social isolation and the absence of supportive relationships (Mallorqui-Bague et al., 2017). Poor sleep hygiene, which exacerbates emotional dysregulation, was also reported (Moreno et al., 2022).

Longitudinal evidence is limited and inconsistent. For example, Wartberg et al. (2023) found that IGD symptoms predicted higher problematic alcohol use at 12-month follow-up, while Borges et al. (2023) did not identify significant associations between IGD and subsequent substance use, The co-occurrence of IGD and substance use has also been linked to poor mental health outcomes, including increased levels of anxiety and depression, further complicating treatment efforts (Gerdner & Hakansson, 2022; Granero et al., 2021).

## Discussion

The primary objective of this scoping review was to map substance use patterns among individuals with IGD/GD. The findings revealed a significant co-occurrence of IGD/GD with the use of substances, most notably alcohol, tobacco, cannabis, and stimulants such as amphetamines. Substance use was also observed, particularly among clinical populations, indicating complex co-occurrence mechanisms. Burleigh et al. (2019) also observed the association between substance use and behavioral addictions, suggesting overlapping psychosocial vulnerabilities. The current review expands on prior research by focusing exclusively on studies utilizing DSM-5 or ICD-11 criteria for IGD/GD. Potential key mechanisms and moderating factors underlying the relationship between IGD/GD and substance use have been identified, including behavioral reinforcement, psychological vulnerabilities, and sociocultural influences.

Both IGD/GD and substance use seem to share behavioral reinforcement mechanisms such as immediate gratification. Engaging in gaming and substance use can serve as negative reinforcement by temporarily alleviating stress, anxiety, and other negative emotional states. These behaviors can also provide positive reinforcement through immediate gratification and pleasurable experiences, such as in-game rewards or substance-induced euphoria (Burleigh, Griffiths, Sumich, Wang, Stavropoulos, et al., 2022; Granero et al., 2021). Both types of reinforcement - negative (e.g., stress avoidance) and positive (e.g., euphoria, gaming rewards) - may contribute to the development of dependency and the maintenance of problematic involvement in IGD/GD (Imataka, Sakuta, Maehashi, & Yoshihara, 2022).

From a neurological perspective, prolonged gaming can disrupt baseline dopamine levels and neural responses, diminishing the brain's sensitivity to natural rewards. This desensitization may heighten vulnerability to substance use as an alternative source of stimulation and reward (Greenfield, 2022). Individuals with addictive disorders often prioritize short-term rewards from gaming and/or substance use over long-term consequences, reinforcing such behaviors (Bickel, Madden, & Petry, 1998). Both disorders involve overlapping pathways in the brain's dopaminergic reward system, where dopamine release enhances reward-seeking and tolerance over time (Ma et al., 2024). This mechanism could be especially important in competitive gaming, where stimulants like caffeine or amphetamines can be used to enhance focus and performance (Ip et al., 2022; Park, Han, Potenza, & Zhao, 2025). The findings suggest potential differences in the neurobiological mechanisms underlying model-based behavior in IGD and AUD (Kwon, Choi, Park, Ahn, & Jung, 2024). Furthermore, from a contextual and cue-reactivity perspective, gaming and substance use may also be maintained by similar environmental cues and triggers, such as stress, boredom, or social pressure (Jang et al., 2022). Beyond these shared triggers, one can hypothesize potential interactions between in-game behaviors and substance use - for instance, using psychoactive substances to enhance focus, prolong gameplay, or intensify the immersive experience of gaming. Such combinations may potentiate the rewarding aspects of gaming (e.g., achievement, escapism), while simultaneously reducing self-regulation and inhibitory control, thereby exacerbating problematic use. To deepen our understanding of these complex dynamics, ecologically grounded research designs - capturing real-time behaviors, contextual influences, and emotional states - are critically needed.

Moreover, emotional dysregulation also emerged as another mechanism for both addictions. Individuals with high levels of neuroticism are more likely to engage in maladaptive behaviors to manage psychological discomfort (Sanchez-Llorens et al., 2021). Poor emotional regulation skills can make individuals rely on external mechanisms, such as gaming or substance use, to alleviate distress (Paulus, Ohmann, Möhler, Plener, & Popow, 2021; Schreiber, Grant, & Odlaug, 2012). Additionally, social isolation, which is a common characteristic in both behaviors, may intensify emotional dysregulation and heighten the risk of maladaptive coping strategies (Matthews et al., 2016). This reliance on external mechanisms can be also reinforced by cognitive biases and expectancies. Cognitive distortions, such as overestimating the benefits of gaming or substances in relieving stress or enhancing social interaction, may contribute to greater dependency on these behaviors. Individuals may also develop expectancies that gaming and substance use will fulfill unmet psychological needs, which reinforces the behaviors (Billieux et al., 2020). Personality and temperament traits, such as impulsivity, novelty-seeking, and sensation-seeking are common risk factors for both IGD and substance use. These traits, commonly associated with ADHD, may exacerbate the development of maladaptive behaviors in gaming and substance use (Ip et al., 2022; Mitchell, Kromash, Holt, & Ginley, 2021).

Furthermore, psychological vulnerabilities play a crucial role in the development and maintenance of both IGD and substance use. These vulnerabilities include difficulties in emotional regulation, impulsivity, sensation seeking, comorbid mental health disorders, and cognitive biases. The reviewed studies also reported that concomitant GD/IGD with other mental health conditions, such as depression, anxiety, and ADHD, may increase vulnerability to IGD and substance use (Granero et al., 2021; Isralowitz et al., 2022). These comorbidities often impair self-regulation, making individuals more vulnerable to the reinforcing effects of both behaviors. Gaming and substances are high-arousal activities that offer immediate rewards, appealing to impulsive individuals (Zuckerman, 1971). Sociocultural influences also play a significant role in IGD/GD and substance use. In certain social circles, particularly among adolescents and young adults, late-night gaming and recreational substance use are normalized or even glamorized (Palma-Ruiz, Torres-Toukoumidis, González-Moreno, & Valles-Baca, 2022). This normalization may lead to sleep deprivation, which in turn exacerbates the engagement in these activities, creating a cyclical pattern difficult to break. Emotional dysregulation caused by lack of sleep may drive individuals toward gaming or substance use, to cope with negative emotions. Cognitive impairments resulting from sleep deprivation, including reduced attention and executive control, further diminish the capacity to resist addictive behaviors (Gujar, Yoo, Hu, & Walker, 2011; Kristensen, Pallesen, King, Hysing, & Erevik, 2021; Wang, Mati, & Cai, 2021; Zhang, Lau, & Hsiao, 2019). Moreover, in some cultures, gaming and substance use are widely accepted as recreational activities, leading to increased exposure and participation (McCauley, Nguyen, McDonald, & Wearing, 2020). Cultural factors were also linked to substance use patterns. Studies conducted in Western countries frequently reported alcohol as a commonly used substance, indicating its cultural acceptance (Borges et al., 2023; Brandao et al., 2022). In contrast, tobacco use was more prevalent in studies conducted in Asian regions, aligning with cultural norms and the availability of smoking in gaming cafes (Liu et al., 2021; Paik et al., 2017). Marketing strategies for games and substances often exploit this acceptance, emphasizing themes of excitement, escape, and achievement. Loot boxes and microtransactions in games mimic gambling mechanics, which are strongly linked to substance use behaviors (Lobstein, Landon, Thornton, & Jernigan, 2017; Thorens, Khan, Khazaal, & Zullino, 2009).

It is also worth noting that while the exclusion of studies using scales published before 2013 and those not based on DSM-5 or ICD-11 criteria brings greater homogeneity to the review, it also resulted in the exclusion of widely used scales such as the Young Internet Addiction Test and GAS-7. Nevertheless, it has been stated that no single scale has been consistently shown to be superior (King et al., 2020). Several limitations in the existing literature were also identified. Most of the studies were cross-sectional and based on self-report assessments only, highlighting the need for more longitudinal and ecological research. Studies have emphasized shared mechanisms between IGD and substance use; however, they do not explore how these mechanisms interact or contribute to the progression of these behaviors over time. There is also a lack of research on protective factors that could mitigate the risks associated with IGD and substance use.

It is important to note that the majority of included studies relied on cross-sectional designs. Consequently, observed relationships between gaming and substance use reflect statistical associations and should not be interpreted as evidence of causal mechanisms. For example, while problematic gaming and alcohol use often co-occurred, it remains unclear whether gaming contributes to increased alcohol use, whether alcohol use influences gaming behavior, or whether both share underlying vulnerability factors such as impulsivity or difficulties in emotion regulation (Müller, Antons, & Brand, 2023; Stellern et al., 2023).

Future studies should explore how media influence, impulsivity, emotional dysregulation, and family dynamics contribute to IGD and substance use. Unstructured parenting (Bussone, Trentini, Tambelli, & Carola, 2020) and stigma-related isolation (Room, 2005) may heighten vulnerability. Sleep deprivation, as both cause and effect, can impair impulse control and heighten reward sensitivity, reinforcing maladaptive behaviors (Burleigh, Griffiths, Sumich, Wang, Stavropoulos, et al., 2022; Granero et al., 2021; Imataka et al., 2022). Research should further examine the bidirectional and temporal links between IGD/GD and substance use, as well as cultural and gender differences in risk and protective factors. Mixed-method, ecological, and multilevel designs can help capture lived experiences and contextual influences. Including individuals with lived experience in intervention design is essential, as are school-based programs promoting emotional regulation in adolescents (Bussone et al., 2020). Moreover, future research should employ longitudinal and ecological momentary assessment (EMA) methods to capture temporal dynamics of gaming and substance use (Zhou, Zhou, Zhou, Shen, & Zhang, 2022). The role of digital environments, such as loot boxes and in-game advertising, should also be examined (Kim et al., 2023; Villalba-García, Griffiths, Demetrovics, & Czakó, 2025). Additionally, cross-cultural studies may clarify sociocultural influences on co-occurring gaming and substance use. Furthermore, when interpreting the current body of evidence, it is important to note that the potential for residual confounding and self-report bias remains, given the reliance on cross-sectional, self-reported data. Nevertheless, overall reporting and measurement rigor across the included studies were consistent with required JBI standards, and the inclusion criteria for eligible studies probably explain the relative homogeneity of the scores observed on some of the quality appraisal items.

From a clinical perspective, these findings underscore the importance of systematically screening for substance use among individuals presenting with symptoms of IGD/GD. Early detection of co-occurring behaviors may help prevent escalation and chronicity. Integrated prevention and intervention strategies that simultaneously target gaming and substance use behaviors could simultaneously enhance treatment outcomes. Such assessments should focus not only on the co-occurrence of both conditions but also on the dynamic interplay between them - for example, the use of cannabis or alcohol during gaming sessions. This approach may facilitate the delivery of tailored cognitive-behavioral interventions and improve psychoeducation on risky digital and substance-related behaviors. Given the high frequency of these concomitant behaviors, close collaboration between addiction specialists, psychiatrists, and clinical psychologists at the organizational level appears essential to ensure interventions are adapted to individual needs.

## Conclusions

The findings revealed that individuals with IGD/GD frequently engage in substance use, including alcohol, tobacco, cannabis, and stimulants such as amphetamines as well as substance use. The relationship between IGD and substance use is complex shaped by various interrelated mechanisms and moderating factors. Based on insights from research and theoretical frameworks, key mechanisms and moderating factors can be identified. These include behavioral reinforcement, psychological vulnerabilities (e.g., coping mechanisms, personality traits, and temperament), co-occurring mental disorders, as well as. sociocultural influences, including the impact of media and peers.

## Appendix

### Bibliographic database search strategies

The research strategies were peer reviewed by a second information specialist prior to execution.

### Medline ALL Ovid

Ovid MEDLINE(R) ALL 1946 to August 08, 2025

limit to yr = “2013–Current”

(exp Substance-Related Disorders/ OR Cannabis/ OR exp Alcohol Drinking/ OR “Recreational Drug Use”/ OR (((substance* OR drug* OR sedative* OR hypnotic* OR anxiolytic* OR psychotrop* OR psychoactive* OR inhalant* OR stimulant* OR hallucinogen*) ADJ3 (use* OR usage OR misuse OR abuse* OR addict* OR depend*)) OR “substance-related disorder*” OR toxicoman* OR cannabis OR marijuana OR marihuana OR hashish OR alcohol* OR ((binge OR problem* OR heavy OR excessive OR underage) ADJ3 drinking) OR tobacco OR smoking OR cocaine OR opiate* OR opioid* OR amphetamine* OR benzodiazepine* OR (solvent* ADJ3 inhala*)).ab,ti,kf.) AND (Video Games/ OR ((video ADJ3 game*) OR videogame* OR videogaming OR ((computer OR online OR “on line”) ADJ3 game*) OR computergame* OR gaming OR “e-sport*” OR esport*).ab,ti,kf.)

958 results on 11.08.2025.

### Embase.com

(‘substance use’/exp OR ‘substance abuse’/exp OR ‘drug use’/de OR ‘recreational drug use’/de OR ‘drug dependence’/exp OR ‘drug abuse’/exp OR ‘cannabis’/exp OR ‘alcohol’/exp OR ‘opiate’/exp OR (((substance* OR drug* OR sedative* OR hypnotic* OR anxiolytic* OR psychotrop* OR psychoactive* OR inhalant* OR stimulant* OR hallucinogen*) NEAR/3 (use* OR usage OR misuse OR abuse* OR addict* OR depend*)) OR “substance-related disorder*” OR toxicoman* OR cannabis OR marijuana OR marihuana OR hashish OR alcohol* OR ((binge OR problem* OR heavy OR excessive OR underage) NEAR/3 drinking) OR tobacco OR smoking OR cocaine OR opiate* OR opioid* OR amphetamine* OR benzodiazepine* OR (solvent* NEAR/3 inhala*)):ab,ti,kw) AND (‘video game’/exp OR ‘game addiction’/exp OR ((video NEAR/3 game*) OR videogame* OR videogaming OR ((computer OR online OR “on line”) NEAR/3 game*) OR computergame* OR gaming OR “e-sport*” OR esport*):ab,ti,kw) AND [2013–2025]/py

1852 results on 11.08.2025

### CINAHL EBSCO

Limit 2013–2025

(MH “Substance Use Disorders+” OR MH “Cannabis” OR MH “Alcohol Drinking+” OR MH “Recreational Drug Use” OR TI (((substance* OR drug* OR sedative* OR hypnotic* OR anxiolytic* OR psychotrop* OR psychoactive* OR inhalant* OR stimulant* OR hallucinogen*) N2 (use* OR usage OR misuse OR abuse* OR addict* OR depend*)) OR “substance-related disorder*” OR toxicoman* OR cannabis OR marijuana OR marihuana OR hashish OR alcohol* OR ((binge OR problem* OR heavy OR excessive OR underage) N2 drinking) OR tobacco OR smoking OR cocaine OR opiate* OR opioid* OR amphetamine* OR benzodiazepine* OR (solvent* N2 inhala*)) OR AB (((substance* OR drug* OR sedative* OR hypnotic* OR anxiolytic* OR psychotrop* OR psychoactive* OR inhalant* OR stimulant* OR hallucinogen*) N2 (use* OR usage OR misuse OR abuse* OR addict* OR depend*)) OR “substance-related disorder*” OR toxicoman* OR cannabis OR marijuana OR marihuana OR hashish OR alcohol* OR ((binge OR problem* OR heavy OR excessive OR underage) N2 drinking) OR tobacco OR smoking OR cocaine OR opiate* OR opioid* OR amphetamine* OR benzodiazepine* OR (solvent* N2 inhala*))) AND (MH “Video Games” OR TI ((video N2 game*) OR videogame* OR videogaming OR ((computer OR online OR “on line”) N2 game*) OR computergame* OR gaming OR “e-sport*” OR esport*) OR AB ((video N2 game*) OR videogame* OR videogaming OR ((computer OR online OR “on line”) N2 game*) OR computergame* OR gaming OR “e-sport*” OR esport*))

388 results on 11.08.2025

### APA PsycInfo Ovid

APA PsycInfo 1806 to January 2025 Week 3

limit to yr = “2013–Current”

(exp “substance use disorder”/ OR exp drug usage/ OR cannabis/ OR (((substance* OR drug* OR sedative* OR hypnotic* OR anxiolytic* OR psychotrop* OR psychoactive* OR inhalant* OR stimulant* OR hallucinogen*) ADJ3 (use* OR usage OR misuse OR abuse* OR addict* OR depend*)) OR “substance-related disorder*” OR toxicoman* OR cannabis OR marijuana OR marihuana OR hashish OR alcohol* OR ((binge OR problem* OR heavy OR excessive OR underage) ADJ3 drinking) OR tobacco OR smoking OR cocaine OR opiate* OR opioid* OR amphetamine* OR benzodiazepine* OR (solvent* ADJ3 inhala*)).mp.) AND (computer games/ OR digital gaming/ OR ((video ADJ3 game*) OR videogame* OR videogaming OR ((computer OR online OR “on line”) ADJ3 game*) OR computergame* OR gaming OR “e-sport*” OR esport*).mp.)

905 results on 11.08.2025

### Cochrane Database of Systematic Reviews Wiley

Issue 8 of 12, August 2025

(((substance* OR drug* OR sedative* OR hypnotic* OR anxiolytic* OR psychotrop* OR psychoactive* OR inhalant* OR stimulant* OR hallucinogen*) NEAR/3 (use* OR usage OR misuse OR abuse* OR addict* OR depend*)) OR (substance-related NEXT disorder*) OR toxicoman* OR cannabis OR marijuana OR marihuana OR hashish OR alcohol* OR ((binge OR problem* OR heavy OR excessive OR underage) NEAR/3 drinking) OR tobacco OR smoking OR cocaine OR opiate* OR opioid* OR amphetamine* OR benzodiazepine* OR (solvent* NEAR/3 inhala*):ab,ti,kw) AND ((video NEAR/3 game*) OR videogame* OR videogaming OR ((computer OR online OR “on line”) NEAR/3 game*) OR computergame* OR gaming OR e-sport* OR esport*):ab,ti,kw

0 results on 11.08.2025

### Cochrane Central Register of Controlled Trials Wiley

Issue 7 of 12, August 2025

Custum range: 2013–2025

(((substance* OR drug* OR sedative* OR hypnotic* OR anxiolytic* OR psychotrop* OR psychoactive* OR inhalant* OR stimulant* OR hallucinogen*) NEAR/3 (use* OR usage OR misuse OR abuse* OR addict* OR depend*)) OR (substance-related NEXT disorder*) OR toxicoman* OR cannabis OR marijuana OR marihuana OR hashish OR alcohol* OR ((binge OR problem* OR heavy OR excessive OR underage) NEAR/3 drinking) OR tobacco OR smoking OR cocaine OR opiate* OR opioid* OR amphetamine* OR benzodiazepine* OR (solvent* NEAR/3 inhala*):ab,ti,kw) AND ((video NEAR/3 game*) OR videogame* OR videogaming OR ((computer OR online OR “on line”) NEAR/3 game*) OR computergame* OR gaming OR e-sport* OR esport*):ab,ti,kw

223 results on 11.08.2025

### Web of Science Core collection

Science Citation Index Expanded (1900-present), Social Sciences Citation Index (1900-present), Arts & Humanities Citation Index (1975-present), Conference Proceedings Citation Index-Science (1990-present), Book Citation Index (2005-present), Emerging Sources Citation Index (2005-present), Current Chemical Reactions and Index Chemicus

Advanced search > More options > Exact search

TS=((((substance* OR drug* OR sedative* OR hypnotic* OR anxiolytic* OR psychotrop* OR psychoactive* OR inhalant* OR stimulant* OR hallucinogen*) NEAR/2 (use* OR usage OR misuse OR abuse* OR addict* OR depend*)) OR “substance-related disorder*” OR toxicoman* OR cannabis OR marijuana OR marihuana OR hashish OR alcohol* OR ((binge OR problem* OR heavy OR excessive OR underage) NEAR/2 drinking) OR tobacco OR smoking OR cocaine OR opiate* OR opioid* OR amphetamine* OR benzodiazepine* OR (solvent* NEAR/2 inhala*)) AND ((video NEAR/2 game*) OR videogame* OR videogaming OR ((computer OR online OR “on line”) NEAR/2 game*) OR computergame* OR gaming OR “e-sport*” OR esport*)) AND PY=(2013–2025)

1655 results on 11.08.2025

### Citation searching

A Backward and Forward citation search was carried out in ***Citationchaser [1]*** on 02.08.2024. Following the detection of an issue in the number of results for the Backward search, it was relaunched in ***ResearchRabbit [2]*** on 14.08.2025.

- *[1] Haddaway, N. R., Grainger, M. J., Gray, C. T. (2021) citationchaser: An R package and Shiny app for forward and backward citations chasing in academic searching. doi:* *10.5281/zenodo.4543513*. *Available from: https://estech.shinyapps.io/citationchaser/*.
- *[2] ResearchRabbit [Internet]. Available from: https://www.researchrabbit.ai*.

### DOIs list of included references:

10.3390/ijerph20032063
10.1080/10550887.2021.1971941
10.1186/s43045-022-00180-6
10.3390/jcm11247370
10.3390/ijerph192316078
10.1016/j.jad.2021.10.064
10.1159/000529544
10.1007/s11469-022-00862-6
10.1186/s43045-022-00219-8
10.1186/s12888-022-04218-1
10.34172/jrhs.2022.91
10.1007/s11469-022-00995-8
10.1007/s10899-021-10079-2
10.1159/000517042
10.1016/j.chb.2021.106890
10.1016/j.addbeh.2020.106432
10.1016/j.adolescence.2021.06.008
10.1556/2006.6.2017.078
10.1007/s11469-019-00100-6
10.1080/14459795.2020.1752768
10.1007/s11469-021-00643-7
10.2196/27719
10.3390/ijerph14121512
10.20882/adicciones.1629
10.1016/j.cegh.2021.100885
10.1080/10826084.2021.1958856
10.1007/s11469-022-00866-2
10.47626/1516-4446-2024-3816
10.1007/978-3-319-41556-7_4
10.1016/j.psychres.2022.114605
10.1016/j.abrep.2022.100406
10.3390/ejihpe15050077

An Update of the database searches identified 4 new references. A Backward and Forward citation search was carried out in ***Web of Science Core Collection*** on 14.08.2025.

### DOIs list of included references:

10.26719/emhj.23.121
10.1556/2006.2024.00045
10.1007/s11469-024-01386-x
10.1016/j.jad.2024.08.203
